# The Neural Correlates of Visuospatial Perceptual and Oculomotor Extrapolation

**DOI:** 10.1371/journal.pone.0009664

**Published:** 2010-03-15

**Authors:** Marc Tibber, Ayse Pinar Saygin, Simon Grant, Dean Melmoth, Geraint Rees, Michael Morgan

**Affiliations:** 1 The Henry Wellcome Research Laboratories, Department of Optometry and Visual Science, City University, London, United Kingdom; 2 Institute of Cognitive Neuroscience, University College London, London, United Kingdom; 3 Wellcome Trust Centre for Neuroimaging, University College London, London, United Kingdom; 4 Department of Cognitive Science, University of California San Diego, San Diego, California, United States of America; University of Leuven, Belgium

## Abstract

The human visual system must perform complex visuospatial extrapolations (VSE) across space and time in order to extract shape and form from the retinal projection of a cluttered visual environment characterized by occluded surfaces and moving objects. Even if we exclude the temporal dimension, for instance when judging whether an extended finger is pointing towards one object or another, the mechanisms of VSE remain opaque. Here we investigated the neural correlates of VSE using functional magnetic resonance imaging in sixteen human observers while they judged the relative position of, or saccaded to, a (virtual) target defined by the extrapolated path of a pointer. Using whole brain and region of interest (ROI) analyses, we compared the brain activity evoked by these VSE tasks to similar control judgements or eye movements made to explicit (dot) targets that did not require extrapolation. The data show that activity in an occipitotemporal region that included the lateral occipital cortex (LOC) was significantly greater during VSE than during control tasks. A similar, though less pronounced, pattern was also evident in regions of the fronto-parietal cortex that included the frontal eye fields. However, none of the ROIs examined exhibited a significant interaction between target type (extrapolated/explicit) and response type (oculomotor/perceptual). These findings are consistent with a close association between visuoperceptual and oculomotor responses, and highlight a critical role for the LOC in the process of VSE.

## Introduction

A major challenge to a comprehensive model of visual perception is how the brain extracts shape and form from the two dimensional retinal projection of a complex and cluttered three-dimensional visual environment. Boundary ownership must be established within the context of objects that are often partially occluded or in motion, and these must be tracked in space and time [1]. The tendency of the visual system to interpolate between fragmented or partially occluded contour elements is one way in which edges and boundaries can be recovered from a scene [2]–[5]. This is thought to be supported by ‘association fields’, which integrate information from spatially separated, but similarly oriented, filter pairs [6]–[10]. However, there is evidence to suggest that a process of visuospatial extrapolation (VSE: see Table 1 for Glossary of Abbreviations) may also be involved in visual completion. Thus, illusory contours may be generated (or distorted) by the co-alignment of an oriented edge with a non-oriented stimulus [11]–[13]. Further, the edges of a partially occluded surface are perceptually elongated beyond the point of occlusion in a process known as boundary extension or amodal continuation [13]–[17].

**Table 1:** Glossary of abbreviations.

**ACW**	Anticlockwise	**pEXP**	Perceptual explicit
**CW**	Clockwise	**pEXT**	Perceptual extrapolated
**d GPrC**	Dorsal pre-central gyrus	**ROI**	Region of interest
**DVA**	Degrees of visual angle	**r GPoC**	Right post-central gyrus
**FEFs**	Frontal eyefields	**r TPJ**	Right temporo-parietal junction
**fMRI**	Functional magnetic resonance imaging	**r VFC**	Right ventro-frontal cortex
**h MT+**	Human motion sensitive middle temporal cortex	**SEF**	Supplementary eyefield
**l GPrC**	Left pre-central gyrus	**SOA**	Stimulus onset asynchrony
**LOC**	Lateral occipital cortex	**v GPrC**	Ventral pre-central gyrus
**mEXP**	Motor explicit	**VSE**	Visuospatial extrapolation
**mEXT**	Motor extrapolated	**VSI**	Visuospatial interpolation
**PEFs**	Parietal eyefields	**V1..5**	Visual area V1..5

In addition to its role in visual completion VSE may also be actively (i.e. voluntarily) initiated by an observer. A multitude of tasks -from basic judgements of stimulus collinearity [18], [19], predictions of occluded line curvature [20], [21] and motion trajectories [22]–[24], to saccades [25], [26] and reaching movements [27], [28] cued by an oriented pointer- require some form of active extrapolation across space, and sometimes time, the underlying neural mechanisms of which are largely unknown at present. These tasks can be carried out over areas of the visual field that exceed the span of known lateral connections in V1 and V2 [29]–[31], suggesting a contribution from higher level cortical processing to VSE.

While there has been a wealth of functional neuroimaging studies of tasks that putatively involve VSE, e.g. modal completion [32]–[37], amodal completion [38]–[42] and contour interpolation [43]–[46], to the authors' knowledge, no single study to date has examined the neural underpinnings of VSE directly using a basic psychophysical task in conjunction with functional magnetic resonance imaging (fMRI). Here, we employed this approach using a block design fMRI paradigm with both whole brain and region of interest (ROI) analyses to identify the neural correlates of VSE and test for the involvement of a number of pre-defined candidate regions.

Sixteen healthy observers were scanned while they judged the relative position of, or actively saccaded to, a target defined by the extrapolated path (in two dimensions) of a pointer. As control tasks, observers made similar judgements and eye movements to explicitly presented (dot) targets. Thus, two factors were independently manipulated in a factorial design; the need for VSE (extrapolated or explicit targets), and the response mode of observers (making a perceptual judgement or initiating a goal-directed saccade). Oculomotor extrapolations were included to distinguish between potentially distinct extrapolation processes for perception and action, which may involve different higher level areas of the ventral and dorsal streams [22], [47]–[49]. Focal analyses were carried out on selected ROIs within the two streams, identified using well-documented localizer tasks. These included the lateral occipital cortex (LOC) [50], which has been implicated in both modal and amodal perceptual completion [41], and components of the eye movement networks that are associated with voluntary saccades and shifts of attention, i.e. the frontal and parietal eyefields (FEFs, PEFs) [51], [52]. The latter were considered relevant as we hypothesized that shifts of attention between the pointer and the extrapolated ‘target’ may actually be critical to the process of VSE [53], [54]. In addition, we also examined activity throughout the brain using a conventional whole-brain random effects analysis. The results we report implicate a role for an occipitotemporal region that includes the lateral occipital cortex (LOC) in both perceptual and oculomotor extrapolations.

## Materials and Methods

Sixteen healthy volunteers aged between 21 and 40 years (7 male) with normal or corrected to normal visual acuity took part in the study. Visual correction was achieved through the use of contact lenses. Fifteen were right-handed by self-report.

### Ethics Statement

All observers gave informed written consent to participate in accordance with the Helsinki Convention and National Institutes of Health guidelines for human subject experiments. The experiment was approved by the Institute of Neurology and National Hospital for Neurology and Neurosurgery Joint Ethics Committee.

### Stimuli

All stimuli were generated in MATLAB (The MathWorks, Natick, MA) using the Psychophysics Toolbox [55], [56] and projected onto a gamma-corrected backlit projection screen (spatial resolution 800×600, temporal resolution 60 Hz). Observers lay supine in the MRI scanner and viewed stimuli at 61 cm via an angled mirror mounted on the head coil.

**Figure 1:**
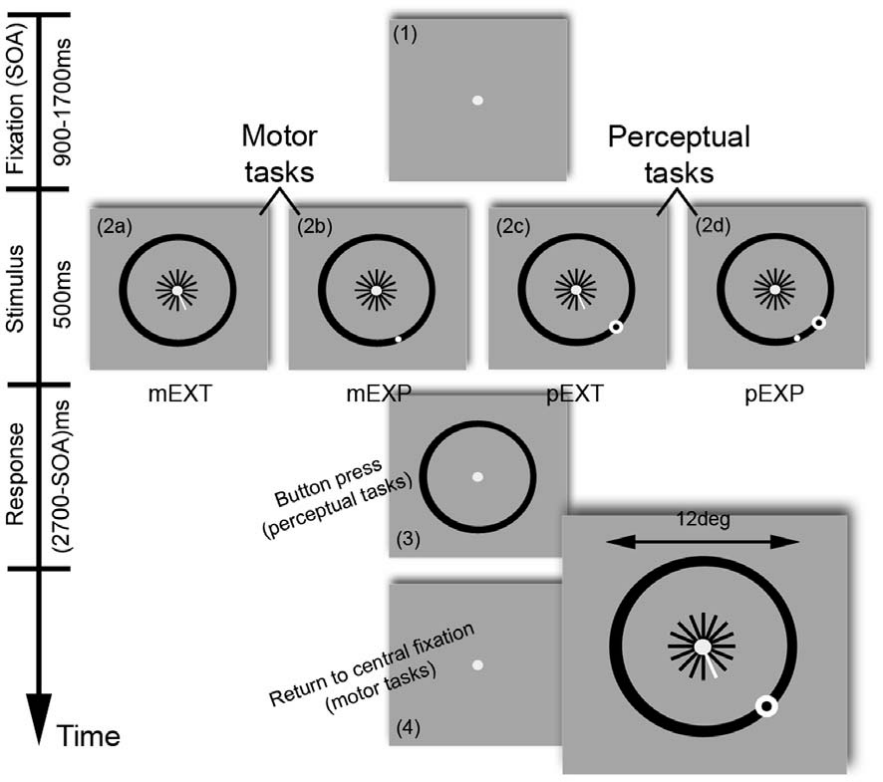
Stimulus presentation sequence. Each trial lasted 3.2 seconds, and began with the presentation of a central fixation point, followed by a stimulus (500 ms) and a variable response period. There were four different experimental conditions, reflecting a 2×2 factorial design [2 factors: response mode (motor or perceptual) and target type (extrapolated or explicit)], in which observers either saccaded to an extrapolated target [motor extrapolated (mEXT)], saccaded to an explicit dot target [motor explicit (mEXP)], judged the offset of a perceptual probe relative to an extrapolated target [perceptual extrapolated (pEXT)] or else judged the offset of a perceptual probe relative to an explicit dot target [perceptual explicit (pEXP)].

The stimulus resembled a wagon wheel with an outer rim radius of 6 degrees of visual angle (DVA) (henceforth referred to as the landing line), and 16 equally spaced spokes radiating from a central fixation point; these subtended one third of the entire radius (see Figure 1). The rim and spokes of the wheel were typically presented in black along with a white central fixation point. In extrapolation conditions (see below), a pointer was defined by making one of the spokes white. In the other conditions, the basic stimulus structure could be augmented by an explicit target (white dot of 0.75DVA diameter), and/or a bulls-eye probe (black dot surrounded by a white annulus 0.2DVA wide), which were superimposed on the landing line. The background grey display was set at a luminance of 2.3 cd/m^2^ and stimulus elements were drawn at maximum contrast.

### Experimental Design – main experiment

A 2×2 factorial design [57] was used in which response mode (motor vs. perceptual; m vs. p) and target-type (extrapolated vs. explicitly defined; EXT vs. EXP) were manipulated. This generated the following 4 conditions, which were presented in a pseudo-random order within a mixed block design paradigm:

1. Motor extrapolated (mEXT) – observers initially fixated centrally and then saccaded to a position on the landing line defined by the orientation of a pointer (extrapolated target).
2. Motor explicit (mEXP) – observers fixated centrally and then saccaded to an explicitly defined (dot) target which appeared on the landing line.
3. Perceptual extrapolated (pEXT) – observers maintained central fixation throughout and judged whether a probe was offset clockwise (CW) or anti-clockwise (ACW) relative to a spatial location on the landing line defined by a pointer (the extrapolated target).
4. Perceptual explicit (pEXP) – observers maintained central fixation throughout and judged whether a probe was offset CW or anti-clockwise ACW relative to the position of an explicit target.

All trials began with the appearance of a centrally located fixation point. Following a variable delay period [a stimulus onset asynchrony (SOA) of 900, 1100, 1300, 1500 or 1700 ms] the entire stimulus was presented for 500 ms; this was then followed by a variable response period (2700 ms – SOA for that particular trial) during which the fixation point and landing line alone remained onscreen, i.e. the spokes and pointer were extinguished. Hence, the overall trial duration was always 3200 ms. In the motor (m) conditions observers were instructed to fixate initially and then saccade to a position on the landing line [explicitly defined by the presence of a target (EXP condition), or defined by the extrapolation of a pointer (EXT condition)] following stimulus onset. Observers then held their gaze on the landing line during the response period and only re-oriented towards the centre with the start of a new trial. In the perceptual (p) conditions observers maintained fixation throughout the trial and indicated by button press whether a probe was offset CW or ACW relative to a target on the landing line (explicit or extrapolated) during the response period. All observers used their right hand to give responses by button press.

When present (EXT conditions), the orientation of the pointer (θ) was randomly sampled from a discrete (integer) uniform distribution of possible orientations ranging from 0 to 180 degrees (measured CW from the horizontal such that 0 degrees represents 3 o'clock on a clock face). The orientations of the non-pointer spokes in the wagon wheel were then calculated relative to the orientation of the pointer (i.e. at a regular spacing of 22.5°). The position of the explicit target (EXP conditions) was sampled from the same discrete uniform distribution on each trial, such that in polar coordinates its eccentricity was defined by the radius of the wagon wheel and the angle theta (θ). Hence, both explicit and extrapolated targets were always presented in the lower hemifield. In the perceptual tasks (p), the position of the bulls-eye probe was always offset by 20 degrees (CW or ACW with equal probability) relative to the position of the explicit or extrapolated target.

### Experimental Design – localizers

In addition to the main experiment separate LOC and eye movement network localizers were acquired from each observer in separate runs. To localize the LOC observers were exposed to alternating blocks of scrambled and unscrambled grey-scale images (800×800 pixels; 14.5DVA×14.5DVA) of recognizable objects taken and adapted from a database provided by Verfaillie and Boutsen [58]. To maintain attentional engagement observers simultaneously performed a one-back-matching task on the images [50]: observers were instructed to maintain fixation on a central dot and press a button whenever two consecutive images were identical. Twenty percent of images presented were followed by a consecutive repeat; these were randomly scattered across trials within a single run. Images were presented on a white background for 200 ms and followed by a blank of 700 ms.

To localize eye movement networks observers performed alternating blocks of endogenously and exogenously cued saccades in a paradigm adapted from that described by Mort et al. [51]. The stimulus consisted of 3 dot targets (1 white and 2 grey; 0.75DVA in diameter) presented against a background black display. The central target was located in the centre of the screen, with flanking dots positioned 6DVA left and right of centre, matching the eccentricity of targets in the main experiment. In the exogenous condition observers made outgoing and return saccades that were triggered by the sequential brightening of a peripheral and central target dot respectively. In the endogenous condition outgoing and return saccades were instead cued by the appearance of an oriented white arrow at the centre followed by a peripheral target location respectively. The direction of the outgoing saccades (left or right) was randomly determined from trial to trial, and the SOA between eye movements was randomly sampled from the following possible durations: 900, 1100, 1300, 1500 and 1700 ms.

### Scanning details

A 3T Siemens Allegra head scanner with standard head coil was used to acquire all functional and structural images. A standard high resolution EPI sequence (matrix 128×128, field of view 192 mm, in-plane resolution 3×3 mm, slice thickness 2 mm with a 1 mm gap, TE 65 ms, TR 2340 ms) was used to acquire 36 slices positioned to enable whole-brain coverage. High resolution T1-weighted structural images (1×1×1 mm) were also acquired.

Each observer completed 6 functional scan runs of the main experiment (6.9 minutes each) in a single scanning session. Each run comprised of experimental blocks (10 trials of a single condition) lasting 32.8 sec each (14 volumes) interleaved with 18.7 seconds of rest (fixation baseline, 8 volumes). Each of the 4 experimental conditions (mEXT, mEXP, pEXT, pEXP) was presented twice in a single run in a counterbalanced order. The order in which the experimental conditions were presented was reordered between scan runs and between observers.

In addition, all observers performed separate runs of an LOC and an eye movement network localizer. These both consisted of 8 experimental blocks (10 volumes/23.4 seconds each) interleaved with 8 blocks of a low-level fixation baseline (7 volumes/16.4 seconds), giving a total run time of 5.3 minutes each. Other scanning parameters were the same as those for the main experiment. An additional 5 volumes (11.7 sec) were acquired at the start of each scan run in all experiments to allow sufficient time for reaching steady state magnetization. This gave a total scanning time (including the main experiment and localizer scans) of approximately 54 minutes. This was spread across a two-hour period that also included observer briefing, the taking of informed written consent and observer de-briefing. Pre-scan offline training of observers was performed previously in a separate, approximately hour-long, session that took place on a different day.

### fMRI data analysis

Image processing and statistical analyses were carried out using SPM5 (The Wellcome Trust Centre for Neuroimaging at UCL, http://www.fil.ion.ucl.ac.uk/spm). The first 5 images from each experimental run were discarded. The remaining images were realigned to the first image to compensate for head movements, spatially normalized to an EPI template provided with SPM-5 (the ICBM-152, as defined by the Montreal Neurological Institute), which closely approximates to the space described by Talairach and Tournoux [59], and spatially smoothed with an isotropic smoothing kernel (7 mm full width at half maximum). A linear combination of regressors representing the time series for each of the 4 experimental conditions (mEXT, mEXP, pEXT, pEXP) and fixation baseline (fixation) were convolved with a synthetic haemodynamic response function and its temporal derivative, creating a box car function. The general linear model was then used to generate parameter estimates of activity at each voxel, for each condition. For the main experiment, a 2^nd^ level (random effects) whole brain analysis was used, for which the contrast images of the four conditions were entered into a 2×2 analysis of variance, using a full factorial model in SPM5. This allowed us to explore both the main effect of target type as well as interactions between the response mode and target type. A threshold of p<0.001, uncorrected for multiple comparisons, was applied to all contrasts. Any inferences drawn could be generalized to the population from which the observers were drawn [60], [61].

Activity within pre-defined ROIs was also examined. The MarsBar toolbox (http://marsbar.sourceforge.net/) was used to extract and average parameter estimates in activated voxels falling within a sphere (radius 5 mm) centered on peak coordinates of activation derived from independent localizer scans (see above). To identify these peaks relevant first level (observer-specific) contrasts were constructed comparing conditions of interest [e.g. non-scrambled > scrambled (LOC localizer) and endogenous > exogenous (eye movement localizer)] for each individual. These first level contrast images were also used to conduct second level group random effects analyses (p<0.001, uncorrected), so that where relevant activations were not evident at the individual level (p<0.001, uncorrected), group level (average) coordinates were used instead. Group level average coordinates were used for 4 out of 16 (4/16) observers for the PEFs, 9/16 observers for the FEFs, 2/16 observers for the SEF and 9/16 observers for the LOC. In addition, we performed an alternative analysis in which we discarded data from observers who did not show activations at the p<0.001 level. Whilst both methods of analysis generated the same pattern of results the effects were somewhat less variable and more pronounced for the larger dataset, presumably because of greater power, which clearly outweighed the small loss of positional specificity. Consequently, only results from the first method of analysis are reported.

For the LOC, peak coordinates in the occipitotemporal cortex were derived from the contrast capturing areas that showed greater activation to non-scrambled images (non-scrambled>scrambled). For the eye movement networks, coordinates for the FEFs and PEFs were derived from activation peaks in the frontal and parietal cortices from the contrast endogenous > exogenous. As the supplementary eyefields (SEFs) were not selectively activated during endogenous saccades [51], a finding that we confirmed, SEF coordinates were gathered from activation peaks derived from the contrast of all eye movements greater than rest [(endogenous + exogenous) > rest]. [Note: henceforth we refer to the supplementary eyefields in the singular form (SEF as opposed to SEFs), as medial activations in the left and right hemispheres associated with the SEFs were found to be contiguous following spatial smoothing]. In addition, as the right temporo-parietal junction (r TPJ) and right ventro-frontal cortex (r VFC) were also clearly highlighted in this contrast [(endogenous + exogenous) > rest], coordinates for these ROIs were also extracted. As with the SEF, these were useful in determining the likelihood that any effects reported were merely driven by attention, as they represent well documented components of attentional networks [62]–[64].

### Eye tracking

Eye movements were recorded throughout scanning sessions using an ASL504 LRO infrared video-based MRI compatible eye tracker (Applied Science Laboratory, Bedford, MA). In addition, observers' eye movements were carefully monitored on a series of video screens, which showed the observers' point of regard superimposed on a view of the stimulus, as well as a direct view of the observers' eye. If appropriate eye movements were not made in the motor task conditions (mEXT and mEXP), or if fixation was not maintained throughout the perceptual task conditions (pEXT, pEXP), the block was terminated prematurely and re-started following renewed instructions to the observer. This only occurred on one occasion.

## Results

### Psychophysical performance and response times in the scanner

**Figure 2:**
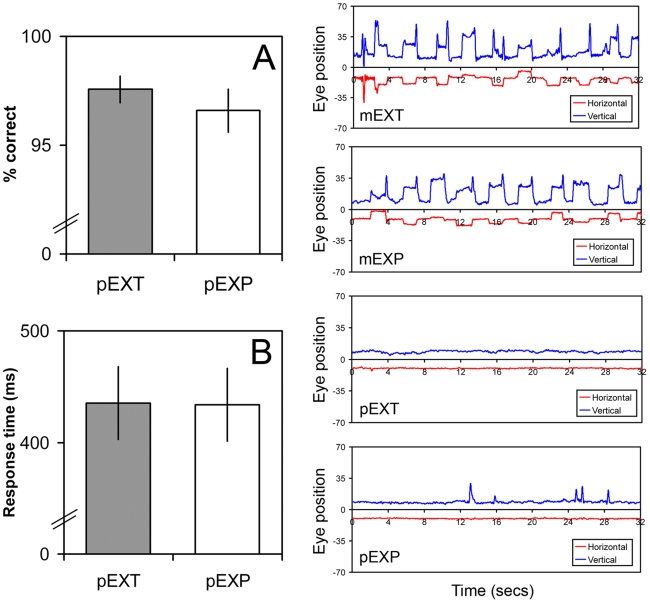
Behavioral data (online). Left panel: (**A**) Percent correct judgements and (**B**) response times are shown for perceptual extrapolated (pEXT) and perceptual explicit (pEXP) task conditions. No statistical differences between conditions were found for either measurement. Right panel: example eye traces for each condition (mEXT, mEXP, pEXT, pEXP) are shown for a single observer. Horizontal (red) and vertical (blue) traces are plotted independently and represent deviations from the mean eye position (fixation) – these have been shifted vertically relative to one another in order that the separate traces may be discerned more clearly. Note the relative lack of eye movements in the two perceptual conditions. Note also that vertical traces in the motor conditions only show positive deflections from the mean; this is because targets were always presented in the lower hemisphere so that the vertical component of all saccades was consistently positive. mEXT – motor extrapolated; mEXP – motor explicit; pEXT – perceptual extrapolated; pEXP – perceptual explicit.

Behavioral data were available for 15 out of the16 observers scanned due to an equipment failure on one occasion. In order to minimize the likelihood that differences in activation patterns between conditions could be due to differences in task difficulty, we deliberately made the discrimination tasks easy to perform; thus, the perceptual probe was offset by 20 degrees, ensuring that performance was ubiquitously high. As shown in Figure 2A, perceptual task performance in the scanner was around 97–98% and did not differ significantly between the two conditions (pEXT vs. pEXP: t_(14)_ = 1.44, P = 0.17; paired-samples t-test). Nonetheless, performance in both tasks was significantly different from ceiling [t_(14)_ = 4, P = 0.001 (pEXT); t_(14)_ = 3.4, P = 0.004 (pEXP); single-sample t-test]. Response times also did not differ between the two perceptual tasks (t_(14)_ = 0.19, P = 0.85; Figure 2B), implying that task difficulty was approximately equal across perceptual conditions.

### Eye movements and fixation

Fixation periods (perceptual and motor tasks) and eye movements (motor tasks) were monitored throughout the experiment. Data on eye position were also recorded. Example eye traces for each condition (mEXT, mEXP, pEXT, pEXP) taken from a single representative observer are shown in Figure 2 (right panel). These were selected solely on the basis that the blocks were comparatively devoid of noise, i.e. there was no prior knowledge of which condition type they constituted. In the motor conditions (mEXT and mEXP) synchronized horizontal and vertical displacements are evident with a characteristic frequency that matches the trial presentation rate (one outbound and one return saccade approximately every 3.2 seconds). Further, these displacements show a boxcar-like profile, indicating that the observers made a saccade and then maintained this peripheral gaze location for a second or two before returning to the centre (as instructed/cued). In contrast, both horizontal and vertical traces show very little deviation from the mean in the two perceptual task conditions (pEXT and pEXP), showing that the observer successfully maintained fixation throughout the block. This pattern was typical of all the observers scanned.

To formalize this difference in behaviour across conditions, eye movement data were also analysed quantitatively. However, as the recordings were often corrupted by noise and gaps in the signal only basic analyses were undertaken. Further, analyses were limited to blocks in which noise was minimal and the signal was relatively continuous. Following temporal smoothing and low pass filtering of the data, all blocks were manually scored as being either usable or unusuable for analysis. Critically however, when this was done, the experimenter was blind to the nature of the block type (motor or perceptual), thus removing the potential to bias the analysis, e.g. by excluding perceptual task blocks in which eye movements were prevalent. A minimum of three usable blocks per task type (motor or perceptual) were deemed necessary for an observer's data to be included in the analysis. However, on average, 25.5 blocks of data were analysed per observer. Consequently, a total of 306 blocks of data taken from 12 of the 16 observers scanned were included in the analysis.

Absolute (unsigned) gaze locations (relative to central fixation; arbitrary units) were compared for motor and perceptual task conditions in a group level paired samples t-test, each pair of entries representing data from a different observer. In both the horizontal and vertical plane, the mean gaze location was more eccentric in the motor task conditions (mEXT, mEXP) than it was in the perceptual tasks (pEXT, pEXP), a difference that was extremely significant (t_(11)_ = 8.58, *P* = 3.35×10^−6^; t_(11)_ = 8.89, *P* = 2.36×10^−6^). Similarly, we examined the standard deviation of the range of gaze locations recorded in the different tasks. These were also far higher in the motor task conditions than in the perceptual task conditions (t_(11)_ = 16.53, *P* = 4.07×10^−9^; t_(11)_ = 14.57, *P* = 1.55×10^−8^). In contrast, the mean gaze location (in both the horizontal and vertical planes) did not differ between extrapolated and explicit conditions; not for the motor tasks (mEXT vs mEXP; t_(11)_ = 1.12, *P* = 0.29; t_(11)_ = 1.64, *P* = 0.13), nor for the perceptual tasks (pEXT vs. pEXP; t_(11)_ = 0.84, *P* = 0.42; t_(11)_ = 0.06, *P* = 0.95). Similarly, the standard deviation of the range of gaze locations did not differ between extrapolated and explicit conditions; not for the motor tasks (mEXT vs. mEXP; t_(11)_ = 1.43, *P* = 0.18; t_(11)_ = 1.7, *P* = 0.12), nor for the perceptual tasks (pEXT vs. pEXP; t_(11)_ = 0.16, *P* = 0.87; t_(11)_ = 0.83, *P* = 0.42). Taken together with the example eye traces provided (Figure 2 right panels), these data support the pattern of behavior we observed whilst monitoring the scanning sessions, and suggest that observers were able to maintain fixation during perceptual task conditions, and made cued systematic eye movements during the motor task blocks. Furthermore, the data suggest that eye movement patterns did not differ between extrapolated and explicit task blocks.

### Whole brain analyses

**Figure 3:**
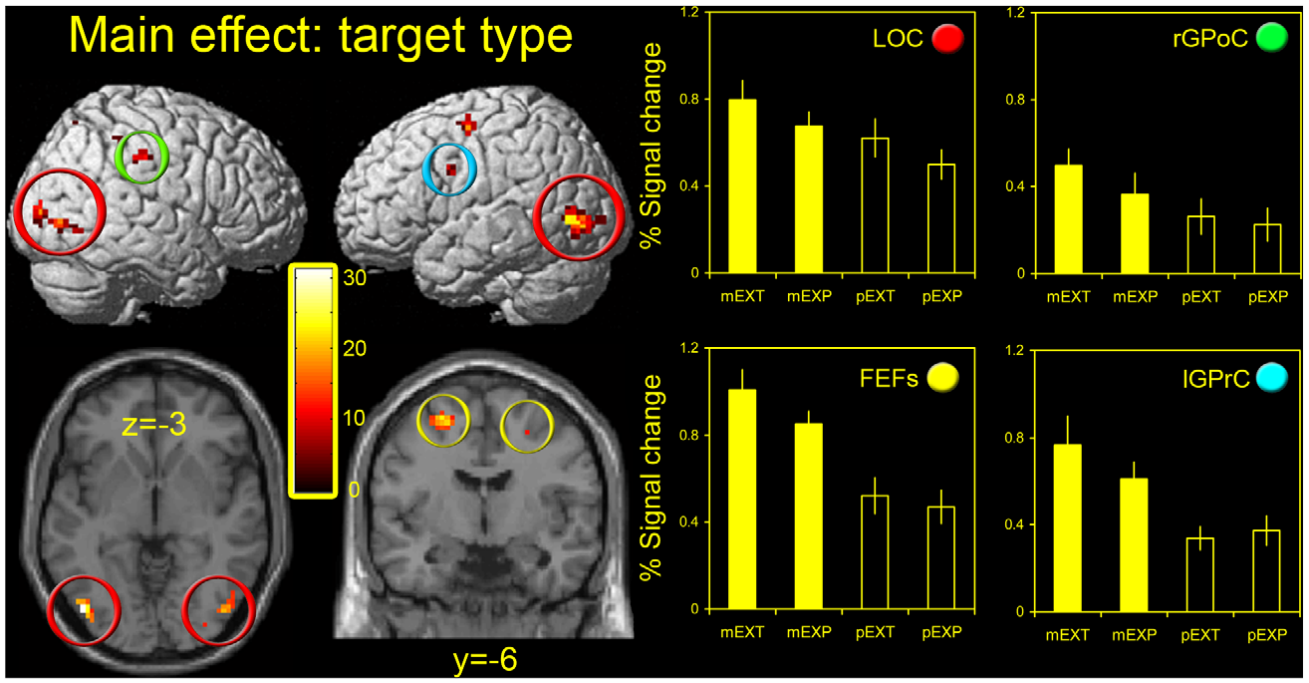
Whole brain analyses – main effect of target type. Two left panels: group level activations are shown for the main effect of target type (*P*<0.001 uncorrected) overlaid on either a T1-weighted structural image rendered in stereotactic space (upper panels) or selected axial and coronal slices (lower panels). In the right panels, estimates of BOLD signal changes from the GLM analysis (relative to rest) for the group of participants are shown for the 4 experimental conditions (mEXT, mEXP, pEXT, pEXP) for the main clusters of activity (two right panels). Error bars represent one standard error of the mean. The colored patches in the upper right corner of the graphs are color coded to match the colored rings in the left hand panels, which denote defined loci in the brain slices and whole brain projections, e.g. red rings illustrate the location of the LOC in the brain slices, and the red circle denotes the accompanying signal plot for that region. LOC – lateral occipital cortex (red rings); FEFs – frontal eyefields (yellow rings); rGPoC – right post-central gyrus (green ring); lGPrC – left pre-central gyrus (blue ring).

Data were initially processed using an exploratory random effects whole-brain analysis (16 observers; *P*<0.001 uncorrected) in order to gain a general impression of the overall pattern of activations. In Figure 3 the main effects of target-type are shown (see Table 2 also). This is a non-directional F-test, and shows areas modulated by target type, irrespective of response mode or the direction of the effects; nonetheless, the direction of the effects can be ascertained from the accompanying activity plots which were derived from 5 mm spheres positioned over local activation peaks. (Note: further statistical analyses of these plots were not performed as the criteria for cluster selection was not independent of the main experiment. This is not true of the ROI analysis, however, as these were identified functionally in separate scan sessions – see next section). Activations were not widespread, but extremely spatially restricted. Thus, clusters of activity were found bilaterally in the occipitotemporal cortex [−45 −78 −3; 45 −78 −3], the coordinates of which are extremely close to those reported for the LOC (see ROI analysis) as well as the adjacent motion sensitive h MT+/V5 complex [65]. In addition, activity was found in bilateral regions of the frontal cortex, which probably relate to the frontal eye fields [27 −9 51; −24 −6 60], as well as restricted foci about the central sulcus [right post-central gyrus (r GPoC) and left pre-central gyrus (l GPrC); 48 −30 39; −54 3 33]. All main effects of target type were clearly driven by greater activation in the extrapolation conditions. No activations were seen in lower level visual areas (i.e. near the occipital pole or in medial occipital locations), nor in the vicinity of the likely locations of the PEFs and SEF.

**Table 2:** Peak activation coordinates - main effect of target type (group data).

Area	x	y	z	*F*	x	y	z	*F*
**LOC**	45	−78	−3	19.3	−45	−78	−3	31.1
**FEFs**	27	−9	51	13.7	−24	−6	60	22.7
**r GPoC**	48	−30	39	18.4	-	-	-	-
**l GPrC**	-	-	-		−54	3	33	16.6

Peak activation coordinates in the stereotactic space of Talairach and Tournoux (x-, y- and z-) and corresponding *F*-values are shown for clusters of activity taken from the main effect of target type in the main experiment. For clusters that were subsequently identified using functional localizers, functional nomenclature is used (LOC – lateral occipital cortex; FEFs – frontal eyefields). For voxels that were not identified functionally, standard anatomical nomenclature is used (rGPoC – right post-central gyrus; lGPrC – left pre-central gyrus).

**Figure 4:**
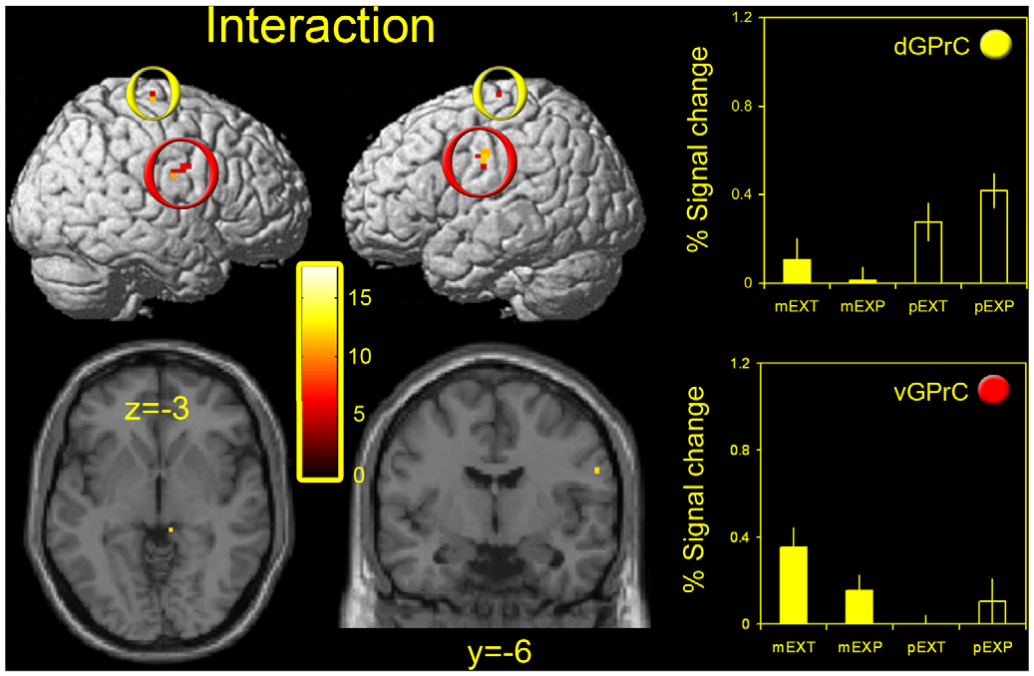
Whole brain analyses – interaction between response mode and target type. Left and central panel: group level activations are shown for the interaction between response mode and target type (*P*<0.001 uncorrected) rendered on a T1-weighted structural image in stereotactic space (upper panels) or selected axial and coronal slices (lower panels). In addition, estimates of BOLD signal changes (relative to rest) from the GLM analysis are shown for the 4 experimental conditions (mEXT, mEXP, pEXT, pEXP) for the main clusters of activity (right panel). Error bars represent one standard error of the mean. As in Figure 3 the colored patches in the upper right corner of the graphs are color coded to match the colored rings denoting defined loci in the brain slices and whole brain projections. dGPrC – dorsal pre-central gyrus (yellow rings); vGPrC – ventral pre-central gyrus (red rings).

Results from the main effects of response mode are not shown as they are largely uninformative with respect to our stated hypotheses. Thus, while activations associated with the main effect of response mode were extensive throughout the brain, this is unsurprising as perceptual and motor task conditions by definition involved very different patterns of foveation and motor activity (i.e. button presses versus eye movements). However, in Figure 4 the interactions between target type and response mode are shown (see Table 3 also). Again, this contrast is non-directional. The first thing to note is the absence of interactions in the occipitotemporal and frontal activations highlighted in the main effect of target type: compare brain slices from Figure 3 and Figure 4, which are taken from identical locations. Thus, activity in these areas was independently modulated by the response mode and target type. In fact, very few areas showed an interaction; activated voxels were largely restricted to bilateral foci in dorsal (d) and ventral (v) regions of the pre-central gyrus [(27 −21 69; −21 −21 72) and (60 −9 24; −54 −12 36) respectively]. Further, activity in these areas was typically relatively low, and in the case of the d GPrC was actually greater in the perceptual task conditions, raising the possibility that there was some interaction between the tasks and response-associated button presses.

**Table 3:** Peak activation coordinates - interaction (group data).

Area	x	y	z	*F*	x	y	z	*F*
**d GPrC**	27	−21	69	14.64	−21	−21	72	12.4
**v GPrC**	60	−9	24	13	−54	−12	36	14.8

Peak activation coordinates (x-, y- and z-) and corresponding *F*-values are shown for clusters of activity taken from the interaction between response mode and target type (main experiment). d GPrC – dorsal pre-central gyrus; v GPrC – ventral pre-central gyrus.

Taken together, data from the whole-brain analyses suggest that VSE is associated with elevated activity in a network of areas that include regions of the ventrotemporal and frontal cortices. However, there is no evidence for interactions between response mode and target type in these areas, and limited evidence for interactions elsewhere in the brain.

### ROI analyses

These effects were probed further, employing a ROI analysis guided by per-participant independent localizers (see Table 4 for group coordinates). To formalize our prior hypotheses, we predicted that the process of VSE would activate the LOC [38], [50], an area that has been implicated in visual completion processes [41]. We also predicted greater activation in areas of the attention/eye movement networks that are specifically involved in the control of endogenously driven shits of attention. These include the PEFs and the FEFs [51], which were identified from the contrast endogenous > exogenous in the eye movement localizer scan. In addition, we identified the SEF from the contrast all eye movements > rest, which we predicted would not be selectively activated during VSE, as it does not show greater activation during endogenous shifts of spatial attention.

**Table 4:** Peak activation coordinates - functional localizers.

Area	x	y	z	*Z*	x	y	z	*Z*
**LOC**	45	−72	3	5.46	−42	−78	−3	5.66
**FEFs**	27	−3	54	4.79	−24	3	57	5.21
**SEF**	-	-	-	-	−9	−3	66	6.09
**PEFs**	27	−75	36	6.56	−21	−69	42	8.32
**rTPJ**	48	−39	12	5.05	-	-	-	-
**rVFC**	57	15	12	5.05	-	-	-	-

Peak activation coordinates (x-, y- and z-) and corresponding *Z*-values are shown for regions of interest defined using functional localizers (group mean data). LOC – lateral occipital cortex; FEFs – frontal eyefields; SEF – supplementary eyefield; PEFs – parietal eyefields; r TPJ – right temporo-parietal junction; r VFC – right ventro-frontal cortex.

**Figure 5:**
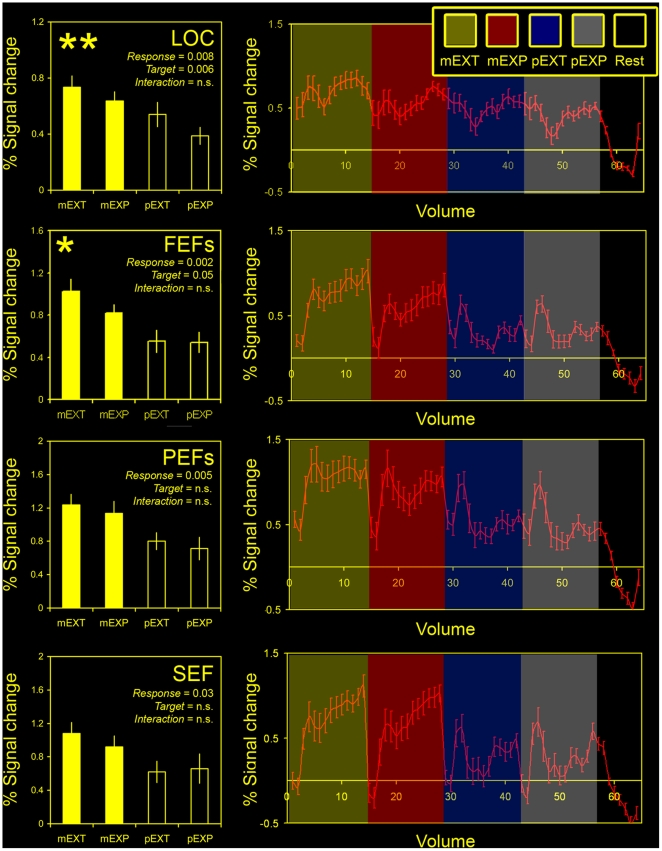
Region of interest (ROI) analyses. The left panel shows estimates of the blood oxygenation level dependent (BOLD) signal changes (relative to rest) from the GLM analysis for the 4 experimental conditions (mEXT, mEXP, pEXT, pEXP) and 4 regions of interest (ROIs). Error bars represent one standard error of the mean. The right panels show corresponding mean BOLD time series plotted for the same ROIs. Data were exposed to a repeated measures ANOVA with two factors (response type: motor or perceptual) and target type (extrapolated or explicit) to test for main effects and interactions. Significant *P* values [main effects of response type (*Response*), target type (*Target*) and interactions between the two (*Interaction*)] are superimposed on the graphs (left panel). Asterisks denote levels of significance for the main effect of target type. *P<0.05, **P<0.01; n.s. – not significant; LOC – lateral occipital cortex; FEFs – frontal eyefields; PEFs – parietal eyefields; SEF – supplementary eyefield; mEXT – motor extrapolated; mEXP – motor explicit; pEXT – perceptual extrapolated; pEXP – perceptual explicit.

Activity in all ROIs was significantly elevated in the motor conditions (Figure 5), reflected by consistent main effects of response mode (Fs_(1,15)_≥5.89,*P*s≤0.03 for all comparisons). This is not surprising, as the motor conditions involved sequential foveation of the distinct stimulus elements. By contrast, only two ROIs examined –the LOC and FEFs- showed a significant effect of target type, supporting the findings of the whole brain analyses. The LOC showed by far the greatest modulation of activity as a function of target type (F_(1,15)_ = 10.21,*P* = 0.006), which was clearly driven by elevations in both extrapolation conditions (i.e. mEXT>mEXP and pEXT>pEXP; see Figure 5 top row). However, there was no significant interaction (F_(1,15)_ = 0.41,*P* = 0.53), suggesting that target type and response mode independently modulated activity in the LOC. In fact, no significant interactions were found in any of the ROIs examined (Fs_(1,15)_≤1.44,*P*s≥0.25 for all comparisons), including the FEFs, in which target type just reached the threshold for significance (F_(1,15)_ = 4.39,*P* = 0.05). The lack of effect of target type in the PEFs or SEF (Fs_(1,15)_≤1.5,*P*s≥0.24) was also shared by other attentional areas: the r TPJ and r VFC (see Figure S1 and Table 4).

Finally, to see if the h MT+/V5 complex [65] could also be involved in the extrapolation process we performed a ROI analysis on observers' data using group average coordinates taken from a previous study designed to locate motion sensitive areas [66] [−44 −70 0; 40 −68 0; Talairach coordinates]. Indeed, main effects of response mode (F_(1,15)_ = 10.45,*P* = 0.006) and target type (F_(1,15)_ = 4.63,*P* = 0.05) were found, raising the possibility that motion sensitive areas also contribute to the process of VSE. However, without functionally localizing h MT+ this remains merely speculative. Nonetheless, it is worth noting that sub-regions of h MT+ have been shown to overlap with the LOC and may exhibit sensitivity to shape [67].

Taken together, data from both the whole brain and ROI analyses suggest that activity is elevated during VSE in the LOC, and to a lesser extent, in the FEFs, with little evidence for an interaction in these areas between target type and response mode. Further, it is possible that there is an additional role for motion sensitive areas in the process, although further data is needed to verify or rule out this possibility.

## Discussion

This is the first study to examine the neural correlates of VSE directly using a basic psychophysical task stripped from the context of shape recognition or contour integration. On the basis of previous studies, we predicted that VSE would activate the LOC [38], [50], which has been shown to play a critical role in visual completion processes [41], as well as regions of the attention/eye movement networks that are specifically involved in the control of voluntary attentional shifts, i.e. the PEFs and the FEFs [51]. In contrast, we predicted no involvement of the SEF, which did not seem to play a specific role in the control of voluntary attentional shifts [51]. Three out of four of these predictions were supported: VSE resulted in greater activation of the LOC, and to a lesser extent the FEFs (relative to carefully matched controlled tasks), but not the SEF. However, contrary to our prediction, we found no evidence for PEF involvement during VSE.

The fact that activity was elevated in specific cortical areas during VSE is consistent with extrapolation tasks involving extra neural processing over and above those involved in the encoding of explicit positional information. However, other explanations should be considered, particularly, the possibility that the extrapolation tasks were more difficult or demanding of attentional resources, resulting in an elevated BOLD signal [68]. Against this, we point out that response times and performance levels in the perceptual tasks (Figure 2) were indistinguishable for the extrapolated and explicit target conditions, strongly suggesting that task difficulty was approximately equivalent. In addition, the main effect of target type was not widespread: VSE resulted in a selective elevation of activity within the LOC, and to a lesser extent within FEFs, but not in other cortical regions involved in visuospatial attention [62] or modulated by attentional load [69], [70]. It is therefore likely that regions implicated during VSE play a more integral role to the process itself.

The finding that the LOC showed the largest effect of target type out of all the ROIs examined is conceptually consistent with existing literature on the role of the occipitotemporal cortex in visual completion processes [32]–[46]. However, in many of these studies the stimuli used were resolvable in terms of real-world phenomena: representations of occluded shapes and objects separated in depth [see Lerner [39] for example], so it is not clear whether the neural correlates implicated were involved in spatial completion processes *per se*, or some later stage of shape identification/object recognition [50] occurring after the constituent contours had been integrated [71]. In contrast, the data reported here suggest a direct role for the LOC in the process of VSE itself. In a related study of visuospatial interpolation (VSI), in which observers had to interpolate between Gabor patches in a three-element alignment judgement, task specific activations (relative to an orientation discrimination control task) were restricted to isolated voxels in locations consistent with previous reports of the LOC [72], [73]. However, it is worth noting that in both these studies a contribution from the adjacent motion sensitive h MT+/V5 complex [65] could not be ruled out. Indeed, recent studies have highlighted some overlap (both functional and anatomical) between the LOC and h MT+ complex, with a subset of motion sensitive voxels exhibiting shape sensitivity, even in the absence of stimulus motion [67].

Results from both the whole brain and ROI analyses also supported our *a priori* prediction of FEF involvement in the process of VSE, although the effect was less pronounced than in the LOC. It is worth noting that whilst the FEFs are associated with attentional effects, they exhibit activity even at relatively low attentional load levels, and further, show little or no additional activity with increasing load [69]. Consequently, even if attentional levels did differ between task types (although this is unlikely given that perceptual task performance was equivalent), this would be unlikely to manifest itself as differential activation in the FEFs. Instead, our rationale for choosing the FEFs as an ROI was that voluntary shifts of attention between the pointer and the extrapolated ‘target’ may actually be critical to the process of VSE. In a dual-task interference paradigm we have previously shown that perceptual extrapolation judgements were disrupted when focal spatial attention was diverted away from the target [54]. Extrapolation of a target's motion has also been shown to involve an active deployment of focal spatial attention along its trajectory [53]. It is therefore worthy of note that activations in the main experiment (main effect of target type) were more extensive in the left hemisphere (Figure 3 and Table 2), a pattern of activity common in studies of endogenously cued shifts of focal attention [see Mort et al. [51] for evidence and a discussion thereof]. What is not clear however, is why, if elevated FEF activation in the VSE conditions reflects the need for voluntary shifts of attention, the PEFs were not similarly implicated. However, subtle differences in experimental design have been shown to modulate the relative contribution of different network components during attentional/eye movement tasks [74]. Neurophysiological and neuroanatomical data on reflexive and voluntary saccades also suggest a more robust association between frontal attentional areas and intentional eye movements [52].

The fact that there was little evidence for an interaction between response mode and target type in the main regions implicated during VSE (i.e. the LOC and FEFs) is consistent with overlapping neural mechanisms of extrapolation for perception and action. In support of this position we have previously shown that perceptual VSE judgements and eye movements share common attentional resources [54], and that pointing movements and perceptual extrapolation judgements are susceptible to a common geometric illusion [27], [75]. While the lack of interaction in our present study was unambiguous in the LOC, the pattern was not so clear cut in the FEFs where the main effect of target type only just reached statistical significance in the ROI analysis, and seemed largely to be driven by differences between mEXT and mEXP task conditions (see Figure 3 and Figure 5). This raises the possibility that some interaction between response mode and target type existed within the FEFs, which we failed to confirm statistically. There is some evidence to suggest the motor system encodes target *motion* independently from perception [22], so that motion extrapolation and 2D spatial (static) extrapolation may represent distinct processes.

There is an alternative interpretation of the data, which is compatible with distinct mechanisms of perceptual and motor extrapolation. Similar activation patterns during motor and perceptual extrapolation tasks may have arisen if observers simultaneously, and in parallel, encoded the extrapolation in both systems whenever a stimulus that afforded VSE was presented, a theory that may be hard to disentangle from premotor theories of visual attention [76], which considers a shift of attention a precursor to, or unexecuted form of, oculomotor plan [77]–[82]. Notwithstanding, the data reported here demonstrate that activity in the FEFs and an occipitotemporal region that includes the LOC is elevated during VSE, and most importantly, whether by virtue of shared neural mechanisms or a tight coupling between action and perception in normal vision, that activity in the LOC is common to both visuospatial perceptual and oculomotor extrapolations. Further studies are needed to resolve these two alternative interpretations of the data (shared mechanisms versus parallel encoding), an undertaking that may shed some light on the basic mechanisms of spatial processing in the context of dual stream models of vision.

## Supporting Information

Figure S1 Ventral attentional network. In addition to the pre-defined regions of interest (ROIs) outlined in the Materials and Methods section, we also examined levels of activity in the right temporo-parietal junction (rTPJ) and right ventro-frontal cortex (rVFC), well established components of the ventral attentional network, which were easily identifiable from the localizer scans within the contrast all eye movements > rest (see Table 4 for coordinates). Superimposed on the graphs are significant P values for the main effects of response mode (Response), target type (Target), and interactions between the two (Interaction). Both regions were modulated by response mode, but neither exhibited a main effect of target type, nor an interaction between target type and response mode. n.s. - not significant; mEXT - motor extrapolated; mEXP - motor explicit; pEXT - perceptual extrapolated; pEXP - perceptual explicit.
(5.45 MB TIF)
