# Supplementary figures and images for: The Neural Correlates of Visuospatial Perceptual and Oculomotor Extrapolation
Source: PLoS One. 2010 Mar 15;5(3):e9664. doi: 10.1371/journal.pone.0009664 (PMC2837745; doi:10.1371/journal.pone.0009664)

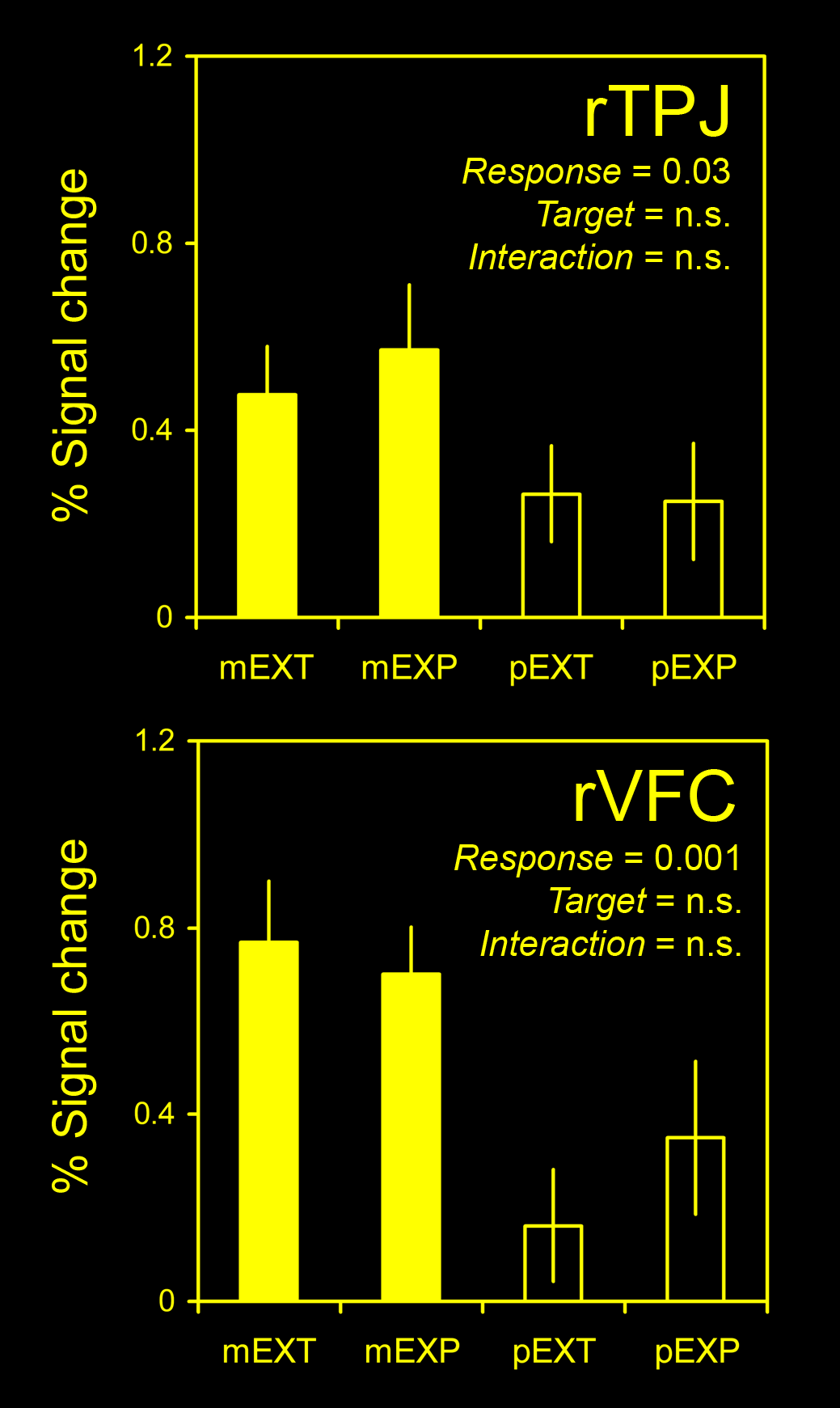

Supplement: Figure S1 — Ventral attentional network. In addition to the pre-defined regions of interest (ROIs) outlined in the Materials and Methods section, we also examined levels of activity in the right temporo-parietal junction (rTPJ) and right ventro-frontal cortex (rVFC), well established components of the ventral attentional network, which were easily identifiable from the localizer scans within the contrast all eye movements > rest (see Table 4 for coordinates). Superimposed on the graphs are significant P values for the main effects of response mode (Response), target type (Target), and interactions between the two (Interaction). Both regions were modulated by response mode, but neither exhibited a main effect of target type, nor an interaction between target type and response mode. n.s. - not significant; mEXT - motor extrapolated; mEXP - motor explicit; pEXT - perceptual extrapolated; pEXP - perceptual explicit. (5.45 MB TIF) [file pone.0009664.s001.tif]
